# Curcumin targets multiple enzymes involved in the ROS metabolic pathway to suppress tumor cell growth

**DOI:** 10.1038/s41598-018-20179-6

**Published:** 2018-02-01

**Authors:** Yonika Arum Larasati, Noriko Yoneda-Kato, Ikuko Nakamae, Takashi Yokoyama, Edy Meiyanto, Jun-ya Kato

**Affiliations:** 1 Laboratory of Tumor Cell Biology, Graduate School of Biological Sciences, Nara Institute of Science and Technology, Ikoma, Nara, 630-0101 Japan; 2 Cancer Chemoprevention Research Center, Faculty of Pharmacy, Universitas Gadjah Mada, Yogyakarta, Indonesia

## Abstract

Curcumin has been reported to exhibit anti-tumorigenic activity; however, since its precise actions remain unclear, its effects are considered to be deceptive. In the present study, we confirmed the anti-tumorigenic effects of curcumin on CML-derived leukemic cells in a xenograft model and *in vitro* culture system. *In vitro* pull-down and mass analyses revealed a series of enzymes (carbonyl reductase, glutathione-S-transferase, glyoxalase, etc.) that function in a reactive oxygen species (ROS) metabolic pathway as curcumin-binding targets, the expression of which was up-regulated in human leukemia. Curcumin increased ROS levels over the threshold in leukemic cells, and the antioxidant, glutathione (GSH) and overexpression of curcumin-binding enzymes partially mitigated the up-regulation of ROS and growth inhibition caused by curcumin. These results show that curcumin specifically inhibits tumor growth by increasing ROS levels over the threshold through the miscellaneous inhibition of ROS metabolic enzymes. Curcumin has potential in therapy to regulate ROS levels in tumor cells, thereby controlling tumor growth.

## Introduction

Tumor cells are generated by multiple mutations in genes that generally function in the growth signaling pathways of mammalian cells, and constitutively-activated, cancer-specific factors are the targets of molecular targeted therapy^1^. In the case of chronic myeloid leukemia (CML), for example, chromosomal translocation t(9;22)(q34;q11) is the leukemia-driving event, which generates the fusion between BCR and ABL genes, and the resultant Bcr-Abl kinase allows cells to survive and proliferate in a growth factor-independent manner^2,3^. The Bcr-Abl kinase-specific inhibitor, imatinib (Glivec, STI571) was found to be very effective and was approved by the FDA as a standard treatment for CML in 2001^4,5^. However, in spite of the use of imatinib as a current first line therapy for CML, its cessation causes relapse in more than 60% of CML patients^6^. The treatment of CML with imatinib leaves residual cells, which are more resistant to imatinib, and may result in the relapse of leukemia. Therefore, in addition to targeting Bcr-Abl, the development of a new approach for the treatment of CML is expected through investigations on other features such as cancer immunology, cancer metabolism, and oxidative stress.

Curcumin is a phytopolyphenol that is mainly found in turmeric (*Curcuma longa*), and has been reported to exert many biological effects including anti-cancer activity on various cancer cells^7,8^. Curcumin has been shown to affect many pathways and factors linked to tumorigenesis^8^, and, despite of its wide range of targets, selectively induces cell death in cancer cells only^9^. However, the precise molecular function of curcumin in tumor suppression remains to be elucidated. Turmeric extracts contain many other compounds besides curcumin^10,11^, suggesting that purified curcumin contains certain contaminants. Furthermore, the stability and pharmacokinetics of curcumin are not optimal^10,11^. Although curcumin is often selected as a positive candidate in many molecular drug screens, its activity is considered to be deceptive^10,11^ partly because of its interference with fluorescent readouts (such as GFP). However 17 recent clinical trials reported efficacy, while another 27 clinical trials and 5 animal studies indicated therapeutic benefits^12^, suggesting that the anti-tumorigenic activity and precise molecular function of curcumin warrant further investigation. In the case of leukemia, curcumin has been shown to down-regulates the Bcr-Abl oncoprotein in CML cells^13^, imitating the action of imatinib; however, the precise molecular actions associated with curcumin have not yet been examined in detail.

In the present study, we reevaluated the anti-tumorigenic activity of curcumin in leukemic cells derived from CML. We used chemically synthesized curcumin rather than that purified from turmeric extracts. We avoided fluorescence (GFP)-dependent assays and, instead, used immunoblots to detect of interactions. We confirmed that curcumin exhibited anti-proliferative activity toward leukemic cells both *in vivo* and *in vitro*. Curcumin specifically bound to and targeted multiple enzymes (carbonyl reductase 1 (CBR1), glutathione-S-transferase phi 1 (GSTP1), aldo-keto reductase family 1 member 1 (AKR1C1), Glyoxalase I (GLO1), NAD(P)H dehydrogenase [quinone] 1 (NQO1), etc) that function in the ROS-metabolic pathway. The treatment of leukemic cells with curcumin increased intracellular ROS levels, and an additional treatment with the strong antioxidant, glutathione (GSH), and the overexpression of curcumin-binding enzymes partially reversed the effects of curcumin. Although the control of intracellular ROS levels is currently one of the most promising therapies for tumor suppression^14,15^, it has not yet been successfully achieved. However, curcumin and its analogues are potential candidates for this purpose.

## Results

### Anti-tumorigenic activity of curcumin in leukemic cells in a xenograft model

**Figure 1:**
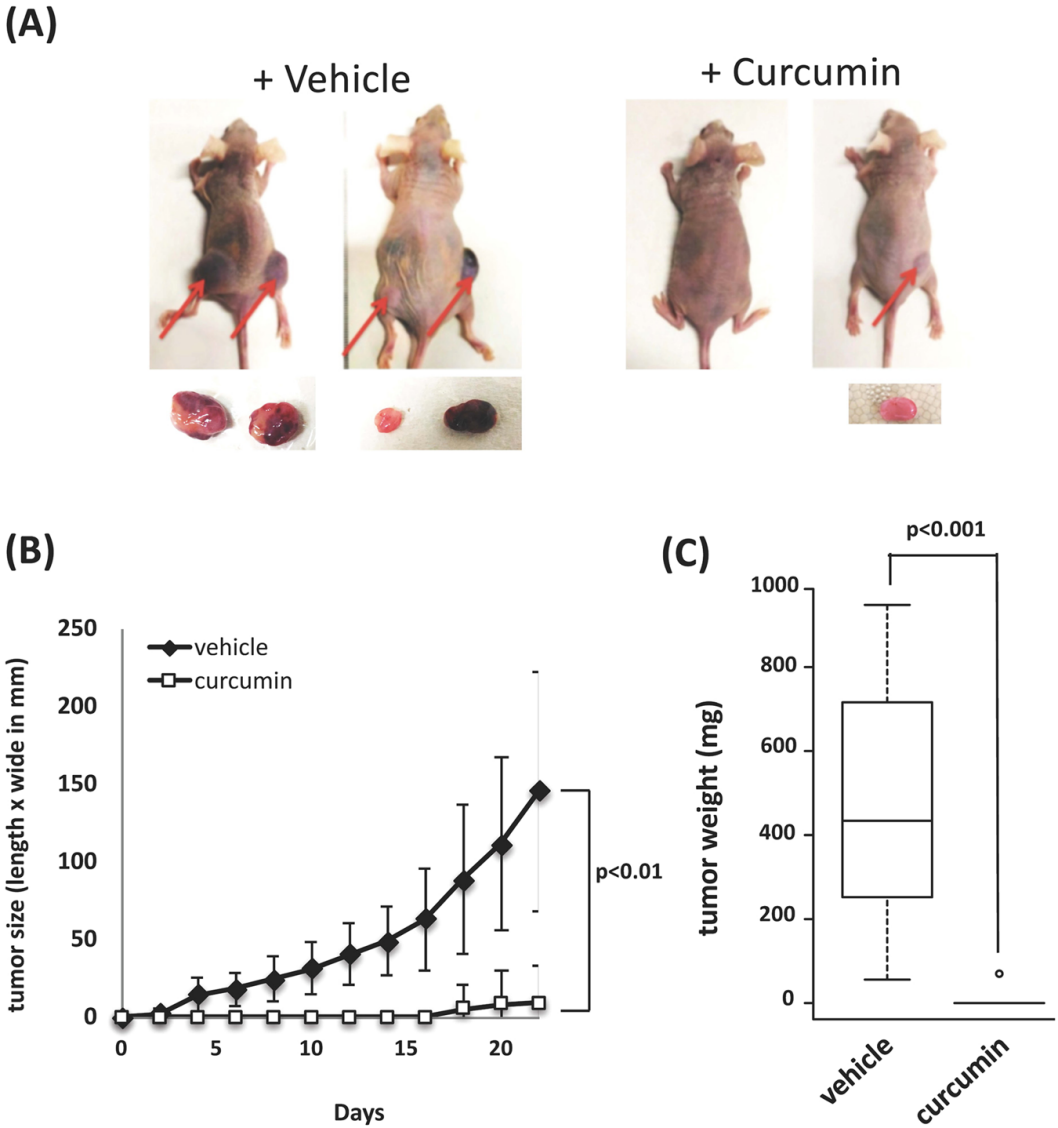
Curcumin suppresses tumor formation *in vivo*. (**A**) K562 cells (2,5 × 10^6^ cells) were transplanted s.c. into the flanks of mice. Mice were then treated with curcumin (25 mg/kg BW) and vehicle (corn oil) via an i.p. injection every two days. After 22 days, mice were sacrificed and tumors were taken. Arrows indicate the tumors formed at the site of injection. (**B**) Tumor sizes (mm) were measured every 2 days. (**C**) Tumor weights after 18–22 days. vehicle (n = 9) and curcumin (n = 8).

Curcumin has been shown to prevent tumorigenic growth in many human cancer cells^7,8^, and we re-evaluated the anti-tumorigenic activity of curcumin in a xenograft model using human erythroleukemic K562 cells, which were derived from a CML patient. K562 cells were subcutaneously (s.c.) injected into the flanks of nude mice (Fig. 1A), and curcumin was then administered to these mice via an intra-peritoneal (i.p) injection every two days for 22 days (Fig. 1B). As a control, corn oil (vehicle) alone was given. Transplanted K562 cells started to form tumors several days after the injection, and these tumors continued to increase in size during the observation period (Fig. 1B, vehicle), whereas no sign of tumors was detected in curcumin-treated mice for 2 weeks after the injection (Fig. 1B, curcumin). After 22 days, visible tumors had formed in all sites of the injection in control mice (Fig. 1A, vehicle), while no or very small tumors were observed in mice treated with curcumin (Fig. 1A, curcumin). A significant difference was noted in tumor sizes (Fig. 1C). Curcumin-treated mice showed no decrease in body weight or other adverse effects in behavior and macroscopic appearance^16^. Thus, curcumin did not induce any obvious adverse effects in the normal lineage of cells, but was sufficiently potent to inhibit tumor progression *in vivo*.

### Curcumin induces irreversible growth inhibition in an *in vitro* culture system

**Figure 2:**
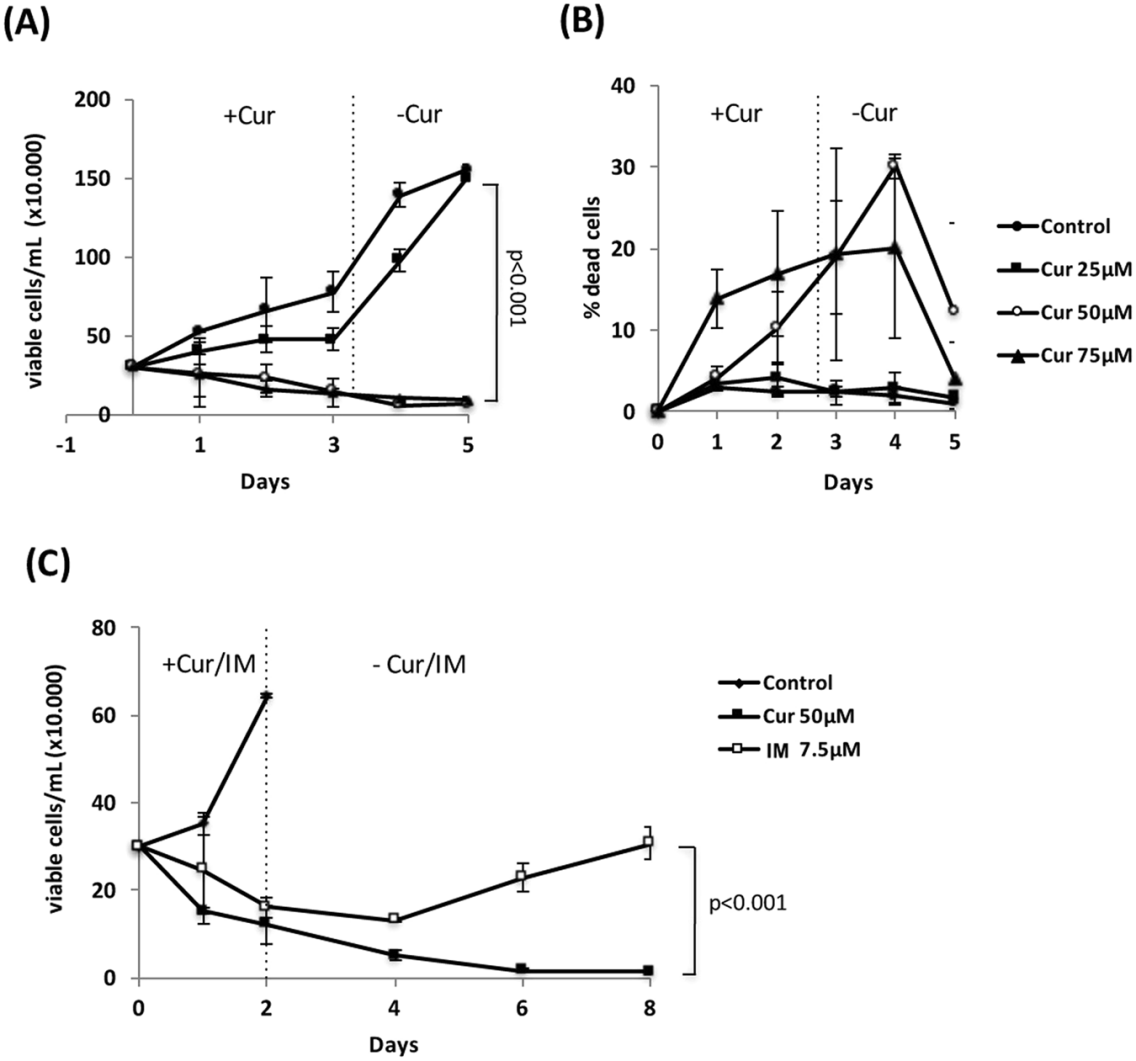
Effects of curcumin and imatinib on the proliferation of K562 cells *in vitro*. (**A**,**B**) K562 cells (3 × 10^5^ cells/mL) were cultured in the presence of curcumin (0, 25, 50, and 75 μM) for 3 days, and curcumin was then removed from the medium. Cells were enumerated every day using the trypan blue staining method. Results for viable (**A**) and dead (**B**) cells are shown. (**C**) K562 cells were cultured in the presence of curcumin (50 μM) and imatinib (IM, 7.5 μM) for 2 days, and the compounds were then removed from the medium. Cells were enumerated at the indicated times. The results are the average of three independent experiments (means ± SD).

In order to further investigate the anti-tumorigenic activity of curcumin, we cultured K562 cells in the absence and presence (25, 50, and 75 μM) of curcumin *in vitro* (Fig. 2A,B). Twenty-five micromolar of curcumin had a negligible effect on the growth of K562 cells, whereas 50 and 75 μM markedly suppressed proliferation. Despite the removal of curcumin from the medium after 3 days, cell proliferation remained suppressed (Fig. 2A). During this period, the percentage of dead cells (estimated using the trypan blue exclusion method) was relatively constant (10–30%) (Fig. 2B), suggesting that some population of cells treated with curcumin was irreversibly growth-arrested, but remained alive. Therefore, we selected 50 μM of curcumin for use in subsequent experiments.

We then compared the growth inhibitory activities of curcumin and imatinib (Fig. 2C). Cells were treated with either compound for 2 days, and then incubated for 6 days in their absence. Curcumin and imatinib both effectively suppressed the proliferation of K562 cells. However, after removal of the compound, cells treated with imatinib started to proliferate again, whereas curcumin-treated cells remained growth-inhibited and gradually lost their viability. These results may reflect the relapse of leukemia reported after the cessation of imatinib^6^, and suggest that curcumin has the good potential for tumor suppression.

**Figure 3:**
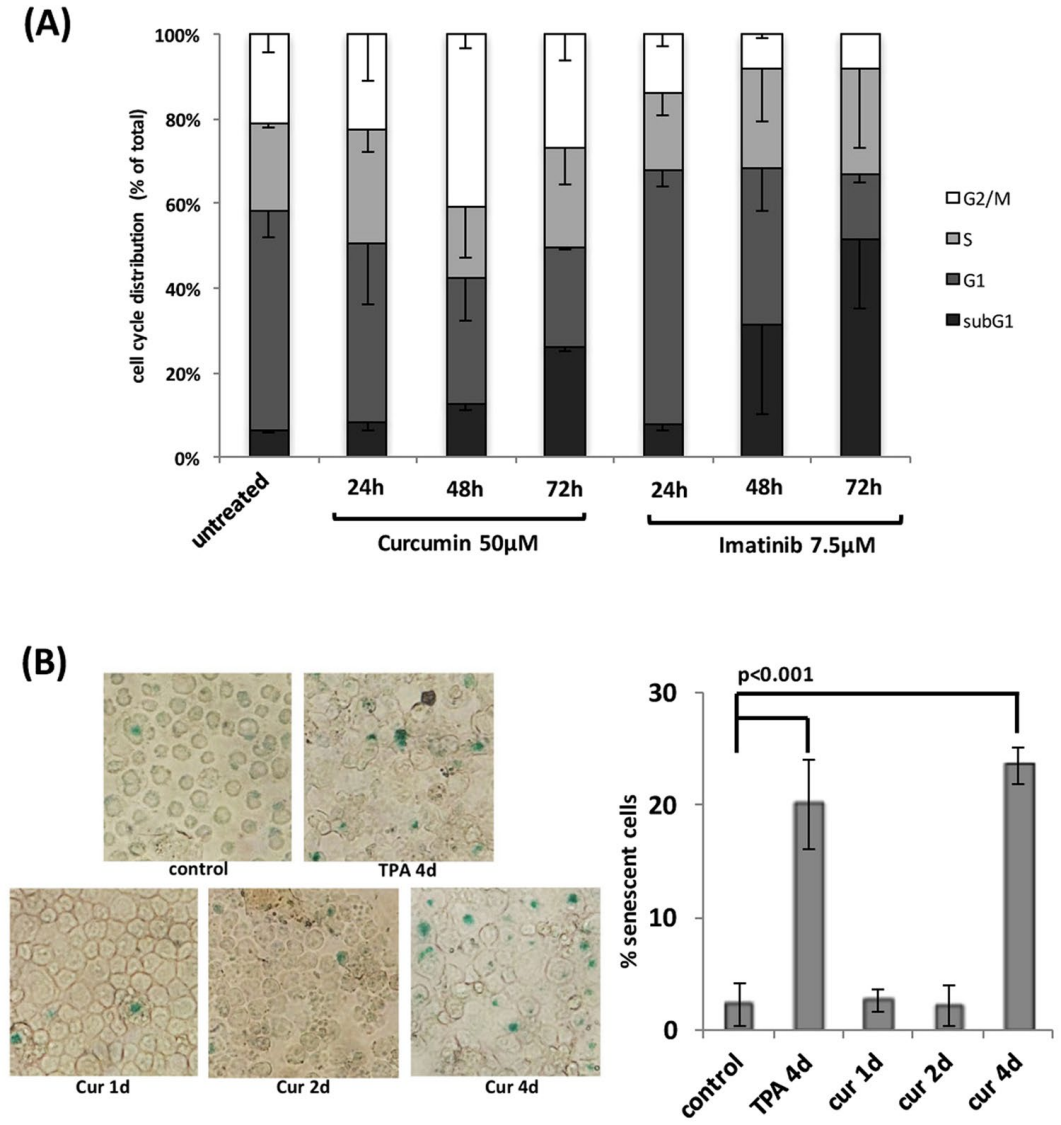
Effects of curcumin on K562 cells *in vitro*. (**A**) K562 cells (5 × 10^5^ cells/mL) were treated with curcumin (50 μM) and imatinib (7.5 μM) for 24, 48, and 72 hours, and then subjected to a cell cycle analysis. (**B**) K562 cells (2 × 10^5^ cells/mL) were treated with curcumin (50 μM) for 1, 2, and 4 days, harvested on the cover glass by cytospin, and subjected to β-galactosidase staining. As a positive control, cells were treated with TPA (16 nM) for 4 days. The percentages of senescent cells (β-galactosidase-positive cells) were calculated (n = 4).

In order to elucidate the anti-tumorigenic activity of curcumin, cells were treated with curcumin and imatinib for 1–3 days and subjected to a cell cycle analysis. Figure 3A shows that cells exposed to imatinib rapidly accumulated in the G1 phase of the cell cycle within 24 hours and subsequently underwent cell death (represented by the subG1 population), while the treatment with curcumin gradually increased the population in the G2/M phase and induced cell death, indicating that the molecular inhibitory actions of these two components differ. Furthermore, the ratio of cell death (represented by the subG1 population) induced by curcumin (ca 25.7 ± 0.7%, subG1 population at 72 hr) was markedly less than that of imatinib (ca. 51.6 ± 16.3%, subG1 population at 72 hr), even though their growth inhibitory effects were the same (Fig. 2C), suggesting an additional anti-proliferative mechanism by curcumin. In Fig. 3B, the SA-β-gal assay revealed that the treatment with curcumin induced cell senescence. Thus, the curcumin treatment induced cell death and senescence, which may be one of the reasons why curcumin so potently inhibited tumor growth.

### Identification of novel curcumin-interacting proteins by an *in vitro* binding assay followed by a mass analysis

**Figure 4:**
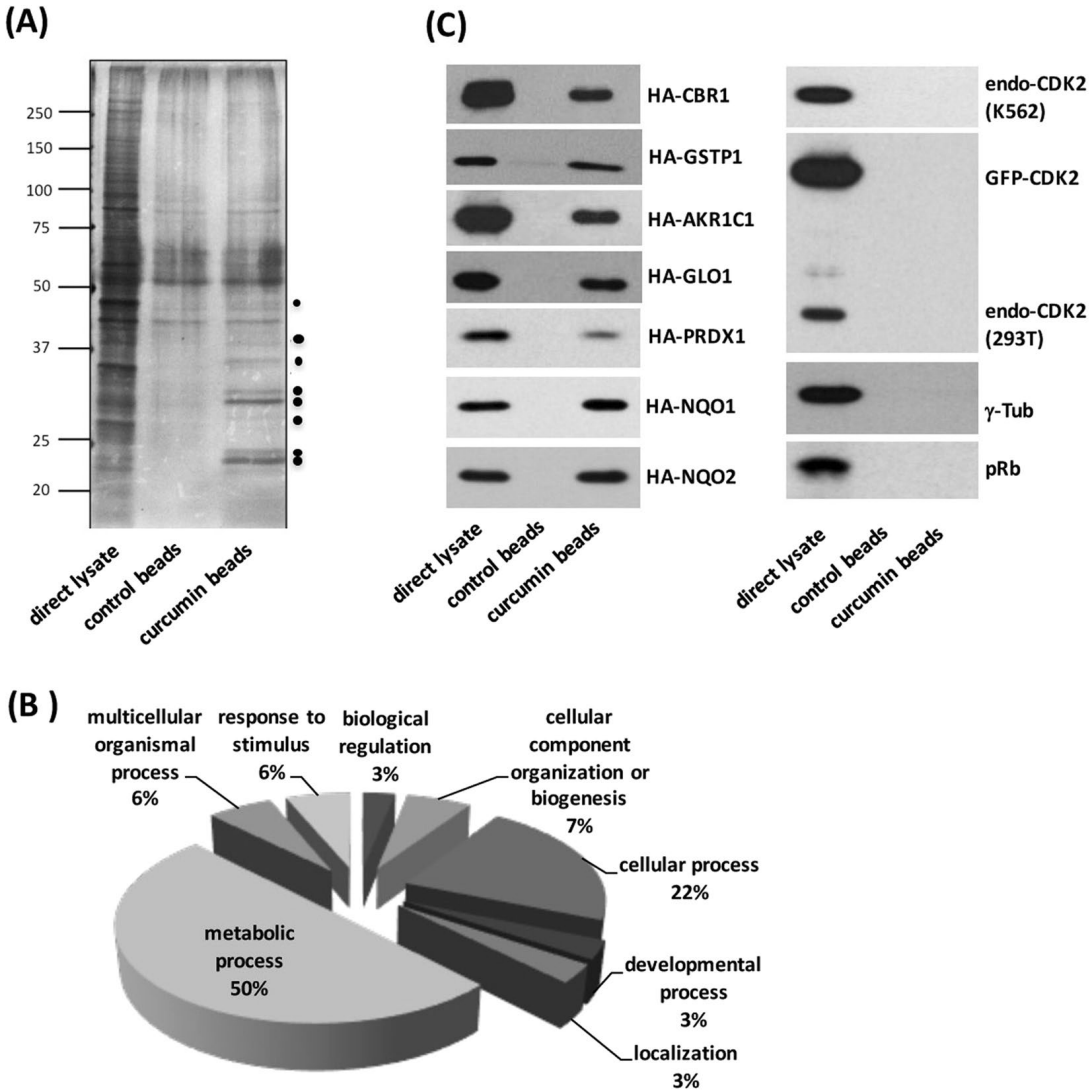
Identification of curcumin-binding proteins in K562 cells. (**A**) The lysate from proliferating K562 cells was incubated with curcumin-sepharose beads (prepared as described in the Materials and Methods). Bound proteins were separated by SDS-PAGE and visualized by silver staining (the bands of putative candidates are marked by the dots). (**B**) Bound proteins were analyzed by MALDI-TOF spectrometry. The list of curcumin-binding proteins (Table 1) was subjected to the PANTHER classification system. (**C**) Lysates isolated from 293 T cells containing HA-CBR1, HA-GSTP1, HA-AKR1C1, HA-GLO1, HA-PRDX1, HA-NQO1, and HA-NQO2 proteins were subjected to the pull-down assay using curcumin beads. Bound proteins were detected by immunoblotting using an anti-HA antibody. Endogenous CDK2, GFP-fused ectopic CDK2, γ-tubulin, and retinoblastoma protein (pRb) were used as negative controls.

In order to elucidate the signaling pathway that curcumin acts on to inhibit leukemic cell growth, we immobilized curcumin on epoxy-sepharose beads^17^ and performed an *in vitro* binding assay using the lysate isolated from proliferating K562 cells. After separation by SDS-PAGE and visualization by silver staining, we identified several bands specific to curcumin beads in the range of 22–45 kDa (Fig. 4A, marked by dots). The portion of the gel corresponding to this region (ca. 20–50 kDa) was digested with trypsin and subjected to a liquid chromatography-mass spectrometry (LC-MS) analysis. After removing the background, we identified 30 candidates as curcumin-specific-binding proteins (Table 1). The classification of curcumin-binding proteins by the PANTHER (Protein ANalysis THrough Evolutionary Relationships) program revealed that half of the candidates were involved in the metabolic process (Fig. 4B), which included carbonyl reductase 1 (CBR1), glutathione-S-transferase phi 1 (GSTP1), aldo-keto reductase family 1 member 1 (AKR1C1), Glyoxalase I (GLO1), NAD(P)H dehydrogenase [quinone] 1 (NQO1), and alcohol dehydrogenase 1 A (ADH1A)^18^. We cloned cDNAs encoding CBR1, GSTP1, AKR1C1, GLO1, PRDX1, NQO1, and NQO2, and expressed them in 293 T cells after HA tagging. We performed a pull-down assay using curcumin beads on lysates isolated from the transfected cells, and found that these proteins were actually present in the curcumin-bound proteins (Fig. 4C). Under these conditions, we did not detect an interaction between curcumin and endogenous CDK2 (cyclin-dependent kinase 2), ectopically-expressed GFP-fused CDK2, γ-tubulin, or retinoblastoma protein (pRb), demonstrating the specificity of the interaction.

**Table 1:** Candidate curcumin-binding proteins.

No.	Protein name	Gene	Theoretical MW (Da)
1.	Carbonyl reductase [NADPH] 1	CBR1	30,461
2.	Glutathione S-transferase P	GSTP1	23,569
3.	NAD(P)H dehydrogenase, quinone 2	NQO2	26,033
4.	Aldo-keto reductase family 1 member C1	AKR1C1	37,221
5.	Biliverdin reductase B	BLVRB	21,960
6.	Carbonyl reductase [NADPH] 3	CBR3	31,230
7.	NAD(P)H dehydrogenase, quinone 1	NQO1	30,905
8.	Sepiapterin reductase	SPR	28,316
9.	Peroxiredoxin-1	PRDX1	22,324
10.	Quinone oxidoreductase	CRYZ	35,356
11.	Glyoxalase I	GLO1	20,861
12.	Beta actin	ACTB	42,128
13.	Ketosamine-3-kinase	FN3KRP	34,618
14.	Aldo-keto reductase family 1 member D1	AKR1D1	37,695
15.	NM23 Nucleoside Diphosphate Kinase 1	NME1	20,740
16.	40 S3 ribosomal protein	RPS3	26,870
17.	Carboxymethylenebutenolidase homolog	CMBL	28,372
18.	Nucleophosmin	NPM1	32,575
19.	Casein kinase II beta subunit	CSNK2B	25,242
20.	Annexin A2 isoform 2	ANXA2	38,808
21.	Glutathione S-transferase omega-1	GSTO1	27,833
22.	40S ribosomal protein S2	RPS2	31,590
23.	dCTP pyrophosphatase 1	DCTPP1	18,783
24.	Ras-related protein Rab-28	RAB28	24,841
25.	Emerin	EMD	29,033
26.	SUB1 homolog, Transcriptional Regulator	SUB1	14,395
27.	Protein-tyrosine-phosphatase, non-receptor type 13	PTPN13	276,915
28.	Proteasome subunit beta type-3	PSMB3	23,219
29.	Alcohol dehydrogenase 1 A	ADH1A	40,745
30.	Enoyl-CoA hydratase 1	ECH1	36,314

### Curcumin increases intracellular ROS levels and a treatment with an antioxidant partially reverses the anti-proliferative effects of curcumin

**Figure 5:**
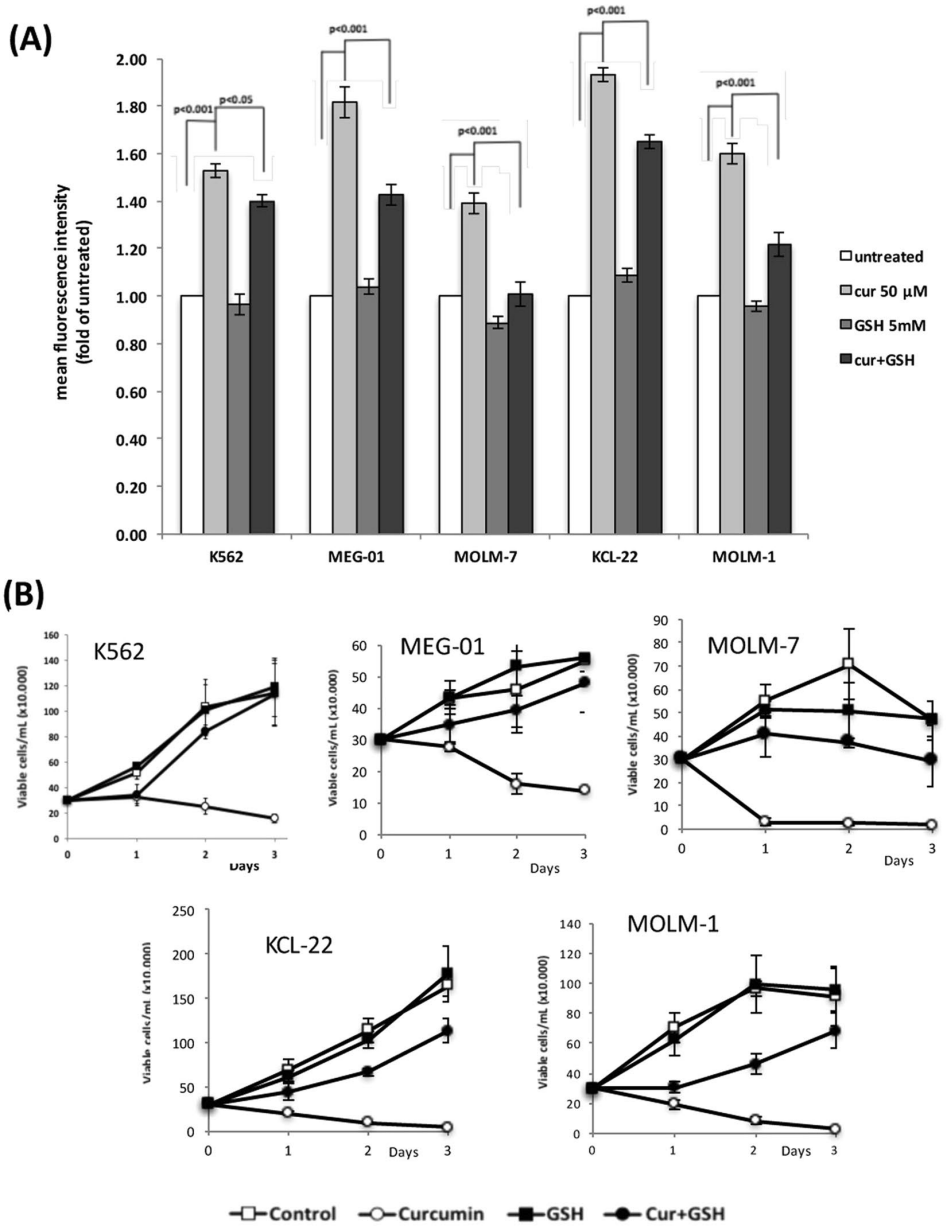
Curcumin increases intracellular ROS levels to inhibit leukemic cell proliferation. (**A**) Cells (5 × 10^5^ cells/mL) were treated with curcumin (50 μM), GSH (5 mM), and both for 24 hours, and subjected to the ROS detection analysis using FACS. (**B**) Cells (3 × 10^5^ cells/mL) were cultured in the presence of curcumin (50 μM), GSH (5 mM), and both, and enumerated every day using the trypan blue staining method. Results are the average of three independent experiments (means ± SD).

Since many curcumin-binding candidates identified in this study (CBR1/3, GSTP1/O1, NQO1/2, AKR1C1/D1, PRDX1, GLO1, and ADH1A shown in Table 1) belong to the enzymes involved in the metabolism of ROS and oxidative-stress products (such as Reactive Carbonyl Species, RCS)^18^, and because curcumin inhibits the enzymatic activity of CBR1^19^, GSTP1^20^, NQO1^21^, and GLO1^22^ *in vitro*, we speculated that the treatment with curcumin up-regulates intracellular ROS levels in CML-derived leukemic cells. CML-derived cell lines (K562, MEG-01, MOLM-7, KCL-22, and MOLM-1 cells) were treated with curcumin for 24 hours, and stained with the fluorescent ROS sensor, Deep Red Fluorescence, followed by a FACS analysis. Figure 5A shows that the curcumin treatment markedly increased ROS levels in leukemic cells. A co-treatment with GSH significantly reduced ROS levels after the treatment with curcumin, while the treatment with GSH had a negligible effect on their levels (Fig. 5A). An incubation in curcumin markedly retarded the proliferation of MEG-01, MOLM-7, KCL-22, and MOLM-1 cells as well as K562 cells (Fig. 5B), indicating that growth inhibition by curcumin is not restricted to K562 cells, but is applicable to other CML-derived cell lines. The additional treatment with GSH of cells that were exposed to curcumin weakened the growth inhibitory effects of curcumin, while the GSH treatment alone had a negligible effect (Fig. 5B). Thus, curcumin increased intracellular ROS levels and inhibited the growth of CML-derived leukemic cells, which was partially rescued by the addition of GSH (Fig. 5A).

### Overexpression of curcumin-interacting proteins partially rescues cells from ROS-mediated anti-proliferative effects of curcumin

**Figure 6:**
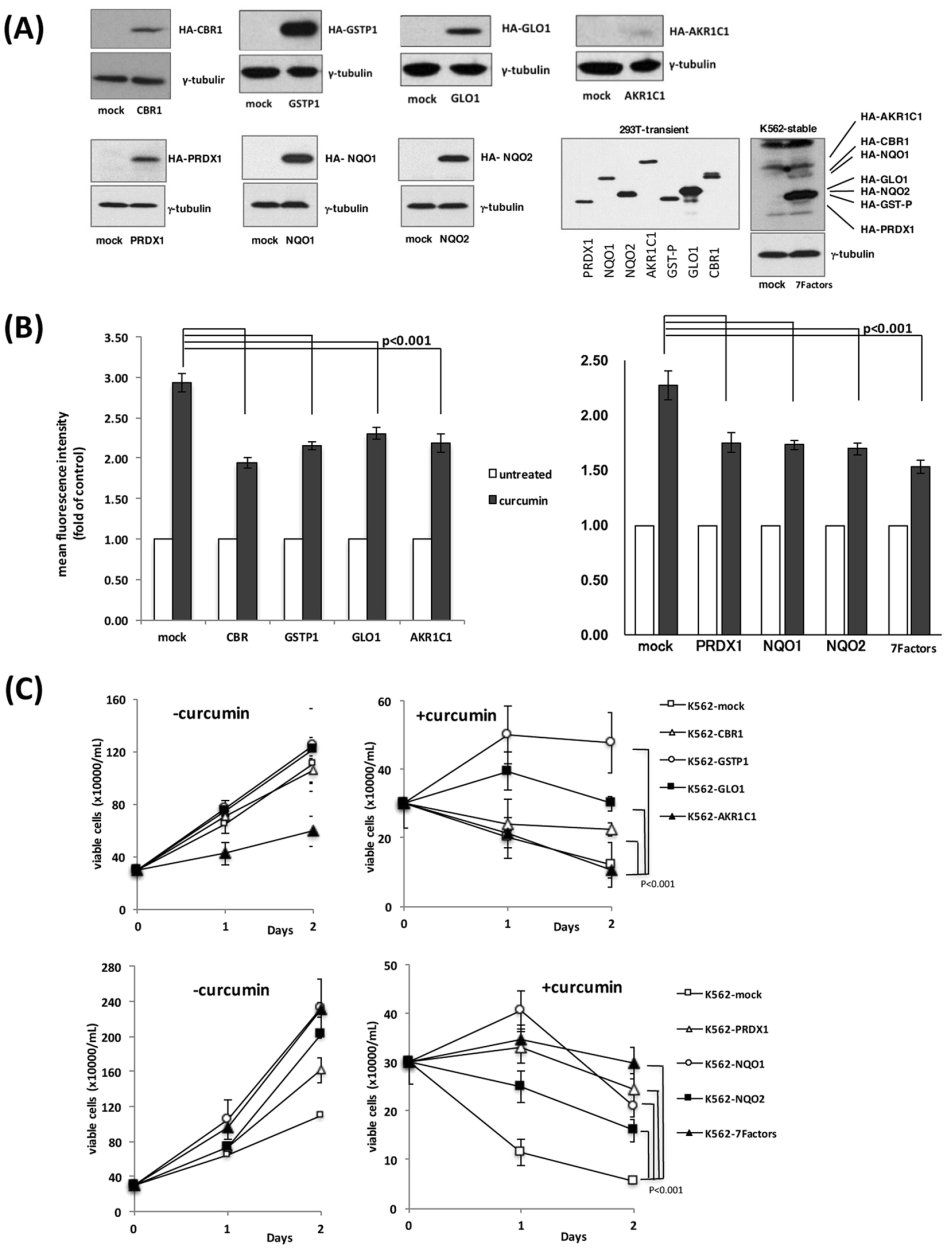
Overexpression of curcumin-binding proteins partially reverses the effects of curcumin. (**A**) Lysates isolated from K562 cells stably introduced with expression vectors containing HA-CBR1, HA-GSTP1, HA-GLO1, HA-AKR1C1, HA-PRDX1, HA-NQO1, and HA-NQO2 cDNAs were subjected to immunoblotting using an anti-HA antibody. K562 cells introduced with all 7 expression vectors were also analyzed. Lysates from 293 T cells transfected with each vector were separately analyzed as a control of the position in the blot. (**B**) Stable K562 cell lines expressing HA-CBR1, HA-GSTP1, HA-GLO1, HA-AKR1C1, HA-PRDX1, HA-NQO1, and HA-NQO2 proteins, and the line introduced with 7 factors were treated with curcumin (50 μM), and subjected to the ROS detection analysis using FACS. (**C**) Stable K562 cell lines expressing HA-CBR1, HA-GSTP1, HA-GLO1, HA-AKR1C1, HA-PRDX1, HA-NQO1, and HA-NQO2 proteins, and the line introduced with 7 factors (3 × 10^5^ cells/mL) were cultured in the presence (+curcumin) and absence (−curcumin) of curcumin (50 μM), and enumerated every day by the trypan blue staining method. Results are the average of three independent experiments (means ± SD).

In order to investigate the role of curcumin-binding enzymes, we introduced HA-tagged expression vectors containing CBR1, GSTP1, GLO1, AKR1C1, PRDX1, NQO1, and NQO2 cDNAs into K562 cells, and established stable cell lines overexpressing these enzymes (Fig. 6A). We also introduced all 7 cDNAs together into K562 cells and isolated the stable line (7Factors) (Fig. 6A, lower right panels). Figure 6B shows that, after the treatment with curcumin, ROS induction levels were markedly lower in overexpressing cell lines than in mock-treated K562 cells, suggesting that the ectopic expression of these enzymes partially counteracted the ROS-increasing effects of curcumin.

The proliferation of cells was similar in overexpressing cell lines and the control line (Fig. 6C, left panels), except that the proliferation of cells overexpressing AKR1C1 was slightly poor. After the addition of curcumin, cells overexpressing CBR1, GSTP1, GLO1, PRDX1, NQO1, and NQO2 (and cells overexpressing 7 factors) tolerated the growth inhibitory effects of curcumin, whereas AKR1C1-cells did not (Fig. 6C, right panels). Since the expression level of the ectopic AKR1C1 protein was not very high (Fig. 6A, upper right panel) and decreased as the cell was maintained in the culture, the overexpression of AKR1C1 may be disadvantageous for cell proliferation. Taken together, we concluded that the overexpression of curcumin-interacting ROS-metabolic enzymes reduced increases in ROS levels and partially rescued cells from curcumin-mediated growth inhibition.

### Curcumin-interacting, ROS-metabolic enzymes are overexpressed in samples from leukemia patients

**Figure 7:**
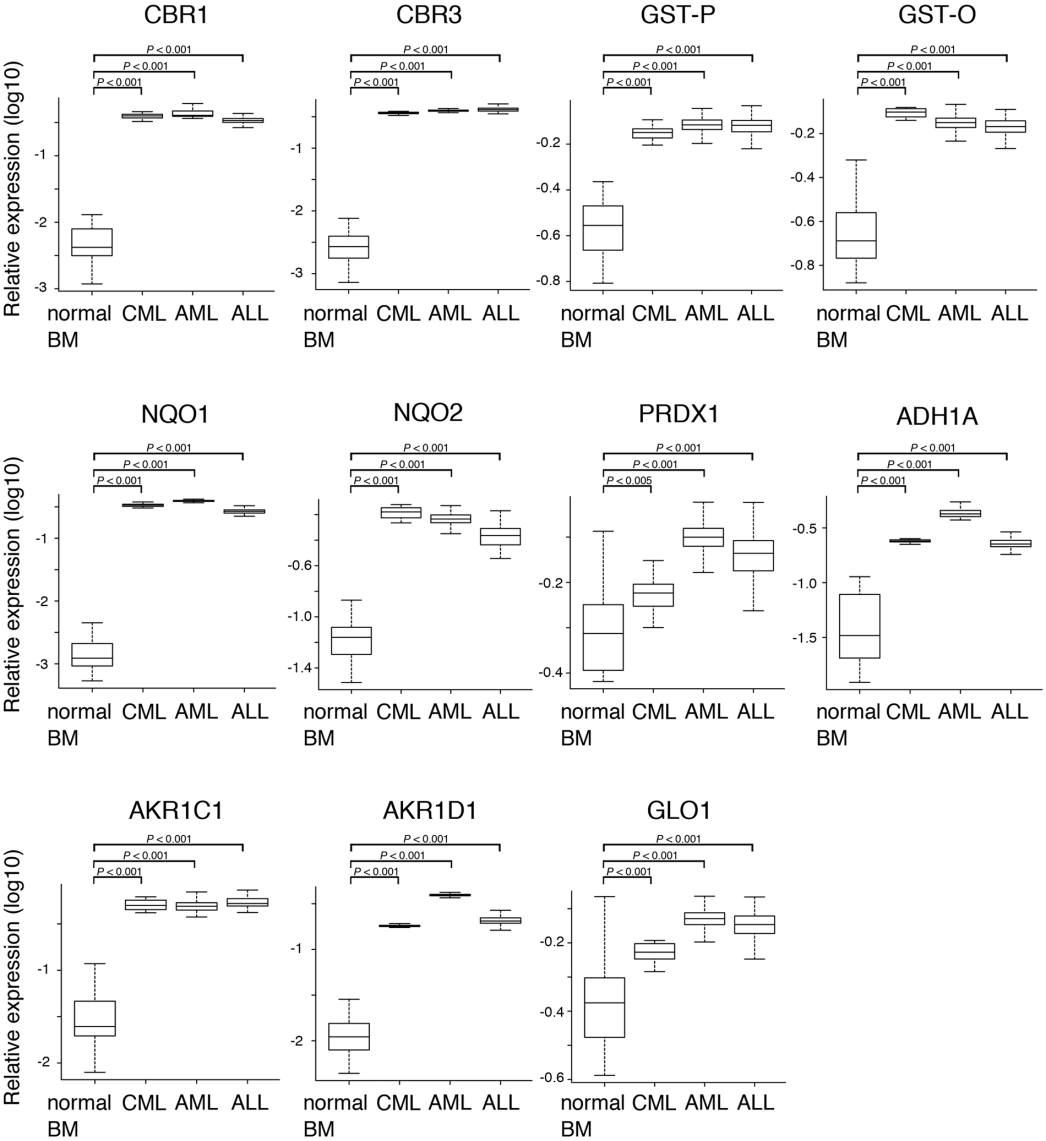
Microarray data analysis of the expression of curcumin interactors in human leukemia. The expression of curcumin interactors (CBR1, CBR3, GST-P, GST-O, NQO1, NQO2, PRDX1, ADH1A, AKR1C1, AKR1D1, and GLO1) in normal bone marrow (n = 22) from healthy donors and CML (n = 18) and AML (n = 461) and T-ALL (n = 100) patients. Gene expression data were obtained from the GEO datasets. Significance was calculated by the Mann-Whitney test.

Since the ectopic expression of curcumin-binding enzymes conferred tolerance to the anti-proliferative signal mediated by ROS, we examined whether these enzymes are up-regulated in human leukemia. We analyzed the expression levels of curcumin interactors (CBR1, CBR3, GST-P, GST-O, NQO1, NQO2, PRDX1, ADH1A, AKR1C1, AKR1D1, and GLO1) in a Gene Expression Omnibus (GEO) dataset of 22 healthy donors, 18 cases of CML, 461 cases of AML, and 100 cases of T-ALL, and found that they were all significantly overexpressed in CML, AML and ALL (mostly *P* < 0.001, except for PRDX1 in CML (*P* < 0.005)) (Fig. 7). These results suggest that curcumin interactors endow some growth advantages to human leukemic cells.

## Discussion

In the present study, we re-evaluated the anti-tumorigenic activity of curcumin using CML-derived leukemic cell lines, the proliferation and survival of which depend on the activity of the oncogenic fusion protein, Bcr-Abl, by comparing it with that of imatinib, a specific inhibitor of Bcr-Abl kinase. We found that imatinib arrested the cell cycle of K562 cells at the G1 phase and rapidly induced apoptosis, whereas curcumin accumulated cells in the G2/M phase and induced apoptosis and senescence. The treatment with imatinib left some residual cells, which were still capable of proliferation after removal of the drug, whereas no cells that had been incubated with curcumin restarted proliferation after its removal. Therefore, curcumin may exhibit superiority over imatinib as an anti-tumor agent.

One of the interesting aspects of curcumin is that it induces apoptosis and senescence, similar to one of the functions of the tumor suppressor p53. However, some cell lines, such as K562 cells, lack functional p53, suggesting that curcumin acts on the checkpoint pathway downstream of or parallel to p53. It is compelling that curcumin acts as a pro-oxidative mediator to induce checkpoint because curcumin is a potent redox scavenging compound. This hypothesis is consistent with our results showing that curcumin binds to several enzymes functioning in the ROS metabolic pathway and regulating intracellular ROS levels, which trigger p53-dependent and -independent checkpoint pathways, resulting in apoptosis or senescence^23–27^.

To date, many proteins have been reported as targets of curcumin^7,8^. In the present study, our binding assay revealed that curcumin interacted with a group of enzymes, including CBR1/3, GSTP1/O1, NQO1/2, AKR1C1/D1, PRDX1, GLO1, and ADH1A, which function in the ROS metabolic pathway^18^. Since curcumin consists of a relatively simple structure, the mechanisms by which it binds to various enzymes need to be elucidated. Since the interaction is specific (curcumin did not bind to CDK2, γ-tubulin or pRb, and exhibited a binding preference to human GST-P and -O, but not to GST from *Schistosoma japonicum*), a specific molecular mechanism appears to exist for curcumin binding to specific target proteins. In addition, the uniqueness of curcumin is that it targets multiple molecules in the same signaling pathway, thereby enhancing inhibition of the pathway. If we successfully elucidate the structural basis for the mechanism by which curcumin simultaneously acts on multiple targets, it will contribute to the development of new types of anti-cancer drugs.

**Figure 8:**
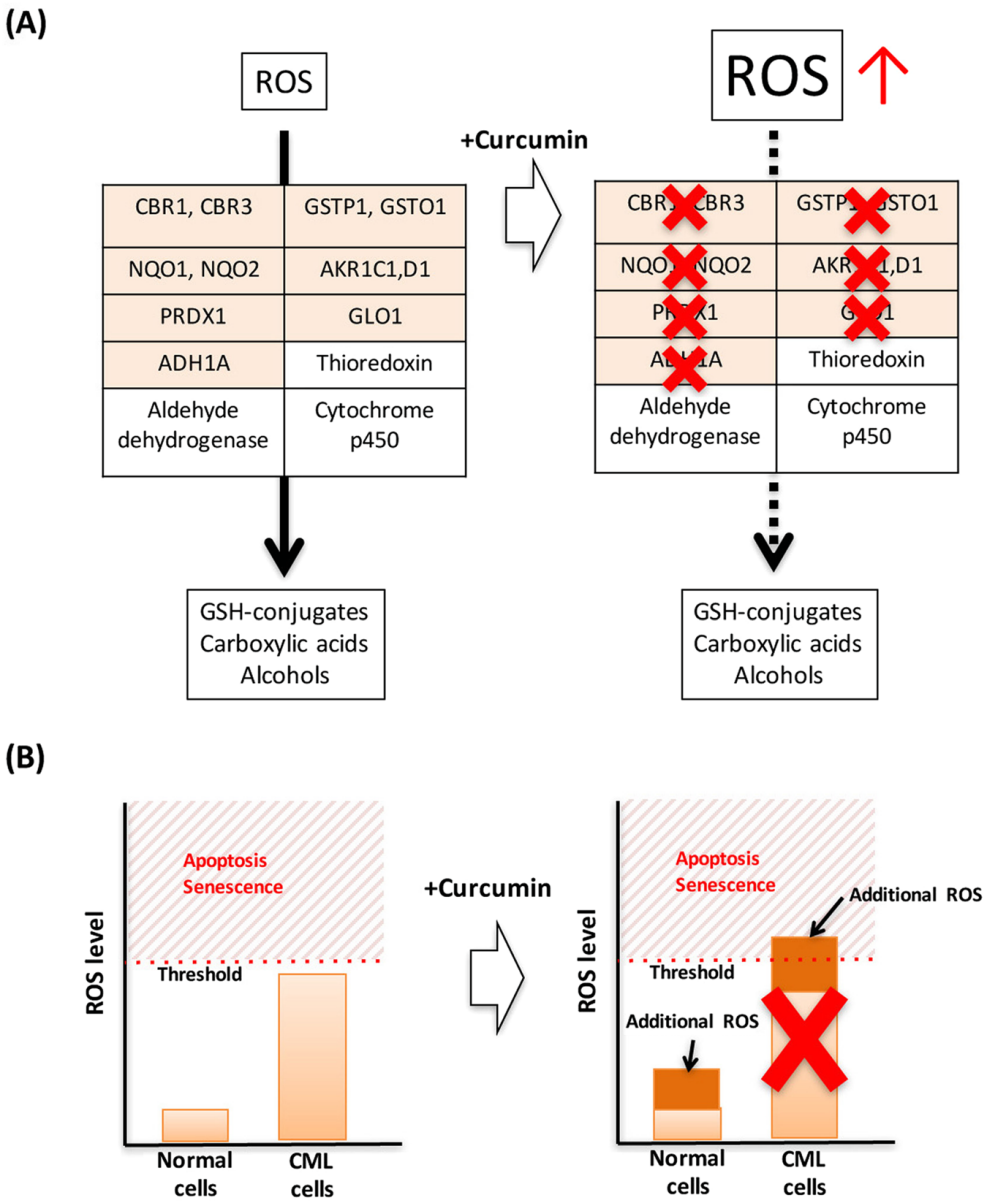
Model of the curcumin-mediated regulation of intracellular ROS (**A**) and cell viability in CML cells (**B**).

Since curcumin binds to several enzymes in the ROS-metabolic pathway and increases the intracellular level of ROS levels (Fig. 8A), and the treatment with GSH weakened the growth inhibitory effects of curcumin, we concluded that curcumin exhibits anti-tumorigenic activity through a ROS-mediated mechanism. However, intracellular ROS levels did not necessarily correlate with the strength of anti-tumorigenic activity, suggesting that the endpoint molecule that actually killed leukemic cells may not be ROS itself, but some other reactive molecules such as reactive carbonyls and reactive aldehydes that are derived from ROS^18^. The identification of these molecules may improve the usage of and modifications to curcumin.

Although curcumin is frequently a positive hit in general drug screens, some people say that it is mostly a false positive^10,11^. This may be partly because curcumin emits fluorescence (similar to GFP), and some screening depends on the same fluorescent signals. However, in the present study, we repeatedly demonstrated that curcumin increased intracellular ROS levels in order to inhibit tumor cell growth. Curcumin is unique in that it targets multiple enzymes, rather than a single protein, in the same pathway with a certain specificity (Fig. 8A). Since basal ROS levels are near the threshold in cancer cells (for CML, see ref.^28^) (Fig. 8B), which is partly due to accelerated proliferation and altered metabolism^25–27^, additional increases in intracellular ROS levels, which are tolerable for normal cells, become lethal to most cancer cells^15,27^ (Fig. 8B). We showed that the effects of curcumin were not restricted to K562 cells, and were extended to other CML-derived cell lines. Based on the nature of the action of curcumin, its anti-tumorigenic effects may be applied to a much wider range of cancer cells. The anti-tumorigenic effects of curcumin have been detected in cancers of the liver, skin, pancreas, prostate, ovary, lung, and head and neck^7^.

Since ROS exert positive and negative effects on cell proliferation and survival, intracellular ROS levels are regulated by multiple factors^25,29^, among which GSH appears to play a major role. Regarding tumor progression, GSH is known to be required together with another antioxidant, thioredoxin for cancer initiation and progression, respectively^30^. Therefore, many attempts have been made to increase intracellular ROS levels in order to suppress tumor development through the inhibition of GSH^31,32^. In the present study, we found that many curcumin-interactor genes were overexpressed in human leukemia samples, CML, AML and ALL, suggesting that tumor cells utilize this pathway to decrease basal intracellular ROS levels. The use of curcumin itself or its derivatives may be a novel approach by which to up-regulate intracellular ROS levels in order to suppress tumorigenesis.

## Materials and Methods

### Curcumin

Chemically synthesized curcumin (the purity is ≥93%) was obtained from the Curcumin Research Center (CRC), Faculty of Pharmacy, Universitas Gadjah Mada, and also purchased from Sigma and Merck.

### Xenograft model (transplantation into nude mice)

Male and female nude mice were housed in specific pathogen-free conditions (the Animal Experimentation Facility of NAIST) in accordance with the NAIST guidelines. K562 cells (2.5 × 10^6^ cells in 100 μl of RPMI-1640 medium supplemented with 10% FBS) were subcutaneously (s.c.) injected into the left and right flanks of 6-week-old nude mice. On the same day (counted as day 0), curcumin (25 mg/kg BW) in corn oil was administered to mice (50 μl of 10 mg/ml curcumin for 20 g BW) via an intra-peritoneal (i.p) injection. The control group received an injection with corn oil alone (vehicle). Curcumin and vehicle were administered every two days for 22 days and tumor sizes were recorded during this period. On the 22^nd^ day, mice were sacrificed, macroscopic appearances were examined, and tumor weights were measured. All methods were carried out in accordance with NAIST guidelines and regulations, and all experimental protocols were approved by the NAIST institutional and licensing committees.

### Cell culture and transfection/electroporation

293 T human embryonic kidney cells were cultured in Dulbecco’s modified Eagle’s medium (DMEM) supplemented with 10% fetal bovine serum (FBS), 2 mM glutamine, 100 units/mL of penicillin, and 100 μg/mL of streptomycin and then transfected with expression vectors via the calcium phosphate-DNA precipitation method^33^.

CML-derived cell lines (K562, MEG-01, MOLM-7, KCL-22, and MOLM-1) were maintained in RPMI-1640 medium containing 2 mM glutamine, 100 units /mL penicillin, 100 μg/mL streptomycin, and 10% fetal bovine serum (FBS). K562 cells were introduced with expression vectors by the electroporation method^34^.

In the growth curve analysis, 3 × 10^5^ cells/mL cells were plated in 35-mm dishes and treated with curcumin, imatinib, and GSH. Where indicated, cells were washed in PBS, and re-plated in fresh medium in the absence of curcumin and imatinib. Viable and dead cells were enumerated using the trypan blue exclusion method.

### Plasmid construction

cDNA fragments containing the entire coding sequences of CBR1, GSTP1, AKR1C1, and GLO1 were amplified by PCR using the K562 cDNA library as a template, and inserted into the HA-tagged expression vector (a gift from Dr. Junichi Fujisawa).

### Cell cycle analysis and β-gal assay

In the cell cycle analysis, cells were stained with 1 mL of a solution of 0.1% sodium citrate and 0.1% Triton X-100 containing 50 μg/mL of propidium iodide and treated with 1 μg/mL of RNase at room temperature for 30 min. Fluorescence from the propidium iodide-DNA complex was measured with a FACSCalibur flow cytometer (Becton Dickinson).

In the senescence associated-β-galactosidase (SA-β-gal) assay, cells were fixed in 0.25% glutaraldehyde, incubated for 16–24 hours in X-Gal solution containing 0.2% X-Gal, 2 mM MgCl_2_, 5 mM K_4_Fe(CN)_6_, and 5 mM K_3_Fe(CN)_6_, and viewed with a phase-contrast microscope.

### Immobilization of curcumin on epoxy-sepharose beads and a pull-down assay

Epoxy-activated sepharose beads (E6754 Sigma) were incubated with 20 mM curcumin in coupling buffer (50% dimethylformamide, 0.1 M Na_2_CO_3_, and 10 mM NaOH) at 37 °C overnight in the dark, and then with 1 M ethanolamine (pH 11) at 37 °C overnight to block the remaining non-specific binding sites. Curcumin-immobilized beads (curcumin beads) were washed with low pH buffer (0.1 M acetate buffer, pH 4, containing 0.5 M NaCl) and high pH buffer (0.1 M Tris-HCl buffer, pH 8, containing 0.5 M NaCl), alternatively, three times, and stored in PBS (pH 7.4) at 4 °C in the dark^17^.

The K562 cell lysate prepared in EBC buffer (50 mM Tris-HCl pH 8.0, 500 mM NaCl, 0.5% NP40) supplemented with 0.1 mM NaF, 0.1 mM Na_3_VO_4_, 10 mM β-glycerophosphate, 1 mM PMSF, and 2 KIU aprotinin was mixed with curcumin beads at 4 °C for 3 h. Bound proteins were eluted from curcumin beads by an incubation with 1.3 mM curcumin solution on ice for 1 h, separated by SDS-PAGE, visualized by silver staining and flamingo staining, and analyzed using MALDI-TOF spectrometry. The list of curcumin-binding proteins was subjected to the PANTHER classification system (http://www.pantherdb.org/).

Alternatively, the cell lysate prepared from 293T cells transfected with HA-tagged expression vectors was mixed with curcumin beads, and the bound protein was analyzed by immunoblotting using a mouse monoclonal antibody to hemagglutinin (HA) peptide epitopes (12CA5, Roche) as described^35^.

### Measurement of intracellular ROS levels

Cells were stained using the Cellular Reactive Oxygen Species Detection Assay Kit (Deep Red Fluorescence, Abcam) according to the manufacturer’s instructions, and analyzed with a FACSCalibur flow cytometer (Becton Dickinson).

### Statistical analysis

Data are presented as the mean ± S.D. and statistical analyses were conducted using SPSS 24. The significance of differences between two experimental conditions was examined using the Student’s *t*-test. The values for the significance of differences were added to every figure.

### Bioinformatics analysis of human gene expression

All human gene expression profiling data were obtained from the NCBI Gene Expression Omnibus (GEO, http://www.ncbi.nlm.nih.gov/geo/). Regarding the relative expression analysis of each gene in human leukemic progression, GEO datasets, accession numbers GSE5900 (normal bone marrows), GSE33075 (CML), GSE14468 (AML), and GSE70536 (T-ALL), were retrieved and analyzed statistically using R version 3.2.2 (R Foundation for Statistical Computing, Vienna, Austria).
